# Challenges in Implementing Cardiovascular Risk Scores for Assessment of Young People With Childhood-Onset Autoimmune Rheumatic Conditions

**DOI:** 10.3389/fmed.2022.814905

**Published:** 2022-02-14

**Authors:** Coziana Ciurtin, George A. Robinson, Ines Pineda-Torra, Elizabeth C. Jury

**Affiliations:** ^1^Centre for Adolescent Rheumatology Research, Division of Medicine, University College London, London, United Kingdom; ^2^Centre for Rheumatology Research, Division of Medicine, University College London, London, United Kingdom; ^3^Centre for Cardiometabolic and Vascular Science, Department of Medicine, University College London, London, United Kingdom

**Keywords:** cardiovascular risk scores, autoimmune rheumatic diseases with childhood onset, young population, cardiovascular risk biomarkers, atherosclerosis

## Abstract

Cardio-vascular risk (CVR) stratification tools have been implemented in clinical practice to guide management decision for primary prevention of cardiovascular disease. Less is known about how we can optimally estimate the CVR in children and adolescents or about the reliability of the risk stratification tools validated in adult populations. Chronic inflammation associated with autoimmune rheumatic disease (ARD) drives an increased risk for accelerated atherosclerosis in patients of all ages. Although the research is less advanced than in adult populations, it is recognized that young people with ARDs with childhood-onset have increased CVR compared to age-matched healthy controls, as supported by studies investigating lipid biomarker profile and markers of endothelial dysfunction. Further research is needed to address the unmet need for adequate CVR identification and management strategies in young people in general, and in those with underlying chronic inflammation in particular. This perspective paper explores various challenges in adequately identifying and managing CVR in younger populations and potential directions for future research.

## Introduction

Ischemic cardiovascular disease (CVD) is an umbrella term which comprises disorders of the heart and blood vessels caused by atherosclerosis, characterized by build-up of lipid deposits within the large and medium arteries leading to increased blood vessel stiffness and impaired blood supply to vital organs, as well as increased risk of blood clots (thrombosis). Atherosclerosis, although it progresses silently over many years, can eventually lead to significant organ damage, such as coronary heart disease, stroke, peripheral arterial disease and aortic disease. The natural evolution of atherosclerotic plaques has been inferred from various studies involving autopsies of individuals of all ages, which in particular revealed the existence of vascular lesions from younger age (1–3). Fatty streaks, defined as the first sign of atherosclerosis visible within the inner layer of blood vessels, start in early childhood. Although some of these arterial deposits can be reversible, they can also progress from an early age to more advanced atherosclerotic lesions through accumulation of lipid-engorged macrophages, T cells recruitment, necrotic core formation due to defective cell death and cellular debris removal mechanisms and development of a fibromuscular cap (4, 5). The existence of early atherosclerotic manifestations in children and adolescents suggests that strategies for cardiovascular risk (CVR) assessment for preventing the development of CVD should start earlier (6). Here we discuss the suitability of using validated CVR stratification tools in younger cohorts, with particular focus on children and adolescents with autoimmune rheumatic diseases (ARDs) who have increased CVR, as well as propose future strategies for improvement of CVR assessment in young patients.

## Markers of Early Atherosclerosis in Children and Adolescents

The presence of atherosclerotic lesions in young people has been detected with high prevalence in various cohorts. Young soldiers who died in the Korean War at a mean age of 22 had evidence of coronary artery atherosclerosis in 70% of cases (7), while more than 50% of children aged 10–14 years killed in road traffic accidents had early atherosclerosis lesions on post-mortem examination (4). Age, in addition to other CVR factors significantly influences the prevalence of atherosclerotic lesions. The large PDAY (Pathobiological Determinants of Atherosclerosis in Youth) study found evidence of aortic atherosclerosis in 20% of healthy subjects aged 14–19 compared to 40% in those age 30–34 (3). In addition, an intravascular ultrasound study detected coronary artery atherosclerosis in 17% of healthy heart donors younger than 20 years, while this proportion increased to 37% in those aged 20–29 and to 60% in adults aged 30–39 years (8). The number and severity of CVR factors, such as increased body mass index (BMI), blood pressure, and levels of serum total cholesterol and low-density lipoprotein cholesterol (LDL-C) all correlated with more severe atherosclerosis lesions in children and young people in the large Bogalusa Heart Study (1). Regarding biological sex, a pro-atherogenic lipid profile has been identified in healthy male adolescents post-puberty, while post-puberal girls had an athero-protective profile when compared to sex-matched prepubertal children (9). This provides evidence that sex-hormones drive changes in lipid metabolism which are relevant for the male-bias in CVD and that these changes start early in life. Lipid abnormalities could be one of the important drivers the driver of the early development of atherosclerotic lesions found in various post-mortem studies, especially as many studies included predominantly young male subjects (10).

## Autoimmune Rheumatic Diseases (ARDs) With Onset in Childhood are Associated With Increased Risk of Atherosclerosis and CVD

Having an inflammatory chronic condition, such as inflammatory arthritis, is associated with increased risk for CVD as well as evidence for accelerated atherosclerosis (11, 12). It is not clearly established how significant the contribution of traditional risk factors, such as age, gender, smoking, or hypertension, are to the development of atherosclerosis in ARD patients (13). Evidence that controlling chronic inflammation associated with ARDs decreases the atherosclerotic risk (14, 15) supports the role of pro-inflammatory cytokines in driving CVR. It is also recognized that autoantibodies frequently present in patients with ARDs could interfere with lipid metabolism (16) and endothelial function (17), therefore contributing to the increased CVR in ARDs. However, there is no current consensus regarding the exact mechanism of accelerated atherosclerosis in ARDs (18).

This is particularly relevant for people with ARDs with childhood-onset because of the longer disease duration and potential long-life exposure to fluctuating chronic inflammation and other detrimental factors. Endothelial dysfunction associated with systemic inflammation represents the first stage in the development of atherosclerosis and can be evaluated through arterial wall dynamic assessments and measurements of intima-media thickness (IMT), which have been shown as being altered in both children and adults with ARDs (19–21).

Juvenile idiopathic arthritis (JIA) is associated with increased prevalence of family history of CVD, hypertension, and smoking, as well as alterations of lipid profile (22, 23). Young adults with JIA have subclinical atherosclerosis even if their arthritis is well-controlled on or off medication (24).

Juvenile systemic lupus erythematosus (JSLE), the prototypical systemic ARD, is associated with a 100- to 300-fold increased mortality from CVD in young patients compared to age-matched controls (25). In addition, JSLE patients are younger when the first CVD event (myocardial infarction) occurs (32.2 years, range 24–43 years) compared with patients with adult-onset SLE (48.1 years, range 19–75 years) (26). Increased CIMT, as marker of subclinical atherosclerosis, has been found to be associated with both traditional and non-traditional CVR factors in a large cohort of JSLE patients included in the APPLE trial of atorvastatin for atherosclerosis prevention (27).

Children with juvenile dermatomyositis (JDM) with a median age of 10, and disease duration of 1.6 years have been shown to already have increased endothelial injury and arterial stiffness, as well as increased markers of inflammation, platelet activation and thrombotic risk compared to age-matched healthy children (28). JDM in children was also associated with premature atherosclerosis reflected in endothelial dysfunction as measured by flow-mediated dilation (FMD) (29). Similarly, young adults with JDM had higher systolic and diastolic blood pressure, increased proinflammatory oxidized high density lipoprotein cholesterol (HDL-cholesterol), IMT and endothelial dysfunction, despite decreased BMI and adiponectin compared to CVD controls (30).

Therefore, there is published evidence that juvenile ARDs are associated with accelerated atherosclerosis and increased CVR factors during both childhood and early adulthood, as well as increased prevalence of both CVR and CVD events in early adulthood.

## What Scores Can We Use to Assess CVR in Younger Adults?

Although significant progress has been made in assessing CVR in the general population for the purpose of primary prevention of CVD, there is less guidance regarding assessment of CVR in younger people with or without associated comorbidities. The Framingham risk score (FRS) was one of the first CVR assessment tools developed in 1998. It uses a gender- tailored algorithm to estimate the 10-year CVR of an individual, and subsequently it is used to guide lifestyle and therapeutic management decisions by identifying the individuals more likely to benefit from such interventions (31). Individuals are arbitrarily grouped in low (<10%), moderate (10–20%), and high (>20%) CVD risk. It has been revised several times, and the most used versions include lipid measurements or BMI (FRS-lipids or FRS-BMI), as BMI has been shown to be an independent predictor of CVR (32). FRS considers traditional CVR factors, such as sex, age, blood pressure, smoking status, and diagnosis of type II diabetes mellitus (which was excluded from the most recent version and replaced with dyslipidemia) (31). As a consequence, although FRS is the oldest and the most widely used CVR stratification tool, it is difficult to appreciate its performance in patients with chronic inflammatory conditions as it does not consider associated CVR burden driven by chronic inflammation. In addition, FRS did not perform well when tested in younger adults (age 18–30), as despite significant CVR burden in some individuals, none of these young people were classified as high risk (33). Despite this, FRS is recommended to be used in individuals older than 20 years of age (34).

More recently, various other CVR assessment instruments have been developed to address the heterogeneity of CVR factors in different populations, by accounting for more CVR determinants, including both modifiable and non-modifiable risk factors, e.g., the atherosclerotic cardiovascular disease (ASCVD) risk score included in the 2019 American College of Cardiology (ACA)/American Heart Association (AHA) guidelines for primary prevention of CVD (35), the QRISK score developed by University of Nottingham and included in the National Institute of Care Excellence guidance for CVD prevention (version 3 the most recent) (36), the Systematic Coronary Risk Evaluation (SCORE) tool developed by the European Heart Association (version 2 the most recent) (37), and the Reynolds Risk Score (RSS) developed in US cohorts in 2007 (38) (Table 1).

**Table 1:** Comparison between various CVR assessment tools.

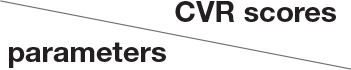	**FRS-lipids/FRS-BMI**	**QRISK3**	**QRISK^®^-lifetime CVR**	**PDAY score**	**ASCVD**	**SCORE 2^#^**	**RSS**
Age	**✓**	**✓**	**✓**	**✓**	**✓**	**✓**	**✓**
Sex	**✓**	**✓**	**✓**	**✓**	**✓**	**✓**	**✓**
Ethnicity/Race			**✓**		**✓**		
Height (cm):			**✓**				
**Clinical information**							
Diabetic?	**✓** **±**	**✓**	**✓**		**✓**		
Previous migraines		**✓**					
Previous CVD events^*^?		**✓**	**✓**				
Angina/heart attack-1st degree relative <60?		**✓**	**✓**				**✓**
Chronic kidney disease (stage 4 or 5)?		**✓**	**✓**				
Atrial fibrillation?		**✓**	**✓**				
On blood pressure treatment?	**✓**	**✓**	**✓**		**✓**		
On statin?					**✓**		
On aspirin?					**✓**		
Rheumatoid arthritis?		**✓**	**✓**				
SLE		**✓**					
hsCRP							**✓**
Severe mental illness		**✓**					
On atypical antipsychotic medication?		**✓**					
On regular steroid tablets?		**✓**					
Diagnosis/treatment for erectile dysfunction		**✓**					
**Modifiable risk factors**							
Smoking	**✓**	**✓**	**✓**	**✓**	**✓**	**✓**	**✓**
Hyperglycaemia				**✓**			
Cholesterol	**✓**				**✓**	**✓**	**✓**
Cholesterol/HDL ratio:		**✓**	**✓**				
HDL—cholesterol	**✓**			**✓**	**✓**	**✓**	**✓**
LDH—cholesterol					**✓**		
Non-HDL-cholesterol				**✓**			
Triglycerides				**✓**			
Systolic blood pressure (mmHg)	**✓**	**✓**	**✓**	**✓**	**✓**	**✓**	**✓**
Diastolic blood pressure (mmHg)				**✓**	**✓**		
SD of at least two recent BP readings (mmHg)		**✓**					
Weight (kg)			**✓**				
BMI	**✓**	**✓**		**✓**			
**Ages for which the score is recommended/has been tested**	20+	40+	30+	15–34 35–54	30+	40+	40+
**Tested in ARDs**	χ, χ~	**✓** ^**^			**✓** ^***^	χ	χ

*ARD, autoimmune rheumatic diseases; BMI, bone mass index; CVR, cardiovascular risk; HDL, high density lipoprotein; hsCRP, high sensitivity C-reactive protein; LDL, low density lipoprotein; SD, standard deviation; SLE, systemic lupus erythematosus*.

# *SCORE-2 is calibrated according to each European country CVD mortality risk*.

± *Excluded from the updated FRS*;

* *heart attack, angina, stroke, or transient ischemic accident*.

** *QRISK2 tested in RA patients aged 40–75 and QRISK3 tested in SLE patients aged 35–44*.

*** *RA patients (40–75 years) classified as moderate/high risk had carotid plaque*.

χ *Underestimated CVR in RA patients*.

χ~ *Performed better in SLE if all items multiplied by 2*.

The ASCVD risk score classified people younger than 40 years of age as low risk in the absence of risk factors (39); however, it is not very clear how well this score performs in younger populations as it has not been tested in children and adolescents. An updated version of the QRISK2 score, aiming at estimating life-long CVR to enable adequate classification of young people, was proposed in 2010—the QRISK^®^-lifetime cardiovascular risk (40). This score had the significant advantage of being able to identify individuals suitable for CVR management interventions at a younger age, with a higher proportion of men, individuals from non-white ethnic groups or with family history of premature coronary heart disease compared to the 10-year estimation of CVR using the QRISK2 score (40).

A systematic review of CVD risk of studies including children age 5–15 revealed that increased BMI significantly worsened other CVR parameters, such as systolic and diastolic blood pressure, serum lipids, fasting insulin, and insulin resistance, suggesting that an adequate CVR stratification tool for young people should take into account these parameters (41). The PDAY study (3) led to the development of a risk score formula to estimate the probability of advanced atherosclerosis in young people age 15-34, using CVD risk factors (42). The PDAY score has been shown to prevent advanced coronary atherosclerosis in both middle-age and young populations (42, 43).

## What CVR Scores Can be Used in Younger Patients With ARDs?

Despite the wide consensus that ARDs are associated with increased CVR (44, 45), and recent initiatives to drive collaborations between Rheumatology and Cardiology to improve the risk stratification of ARD patients (46), there is a paucity of data regarding the performance of various CVR assessment tools in ARD cohorts of all ages. A recent survey of Italian rheumatologists revealed that 67.2% rheumatologists routinely assess the CVR in their patients, while only 18.6% declared that they were managing the patients' CVR themselves, and 50% refer them to other specialties and 23.4% to the general practitioners (47).

Two of the CVR scores described above (Table 1) take into consideration additional clinical information contributing to CVR burden relevant for patients with ARDs, such as a previous diagnosis of rheumatoid arthritis (RA) or systemic lupus erythematosus (SLE) and concomitant steroid treatment (the QRISK3 and QRISK-lifetime scores) (36) and only one includes the high sensitivity C-reactive protein (hsCRP) levels (RSS) (38).

In a large cohort of 31,366 adult patients with RA, there were 1,648 CVD events over a median period of 4 years and the higher ASCVD risk score was associated with the male sex, older age, presence of comorbidities, worse disability, prior fracture, higher disease activity, and glucocorticoid use (48), suggesting that CVR assessment for primary prevention of CVD using validated tools are more tailored to detect CVR in older age. A large study including 1,050 RA patients also found that FRS, RRS, and SCORE all underestimated CVR associated with RA, while the QRISK2 score tended to overestimate it (49). A inception cohort study of 500 RA patients followed up for 8 years found that the observed CVR was higher than predicted by both FRS and RSS (50). The QRISK^®^-lifetime cardiovascular risk also classified inaccurately the CVR of middle-aged males with RA associated with chronic kidney disease as low risk (51), in keeping with the guideline recommendation for its use in younger, female populations (52).

Another study, published only as an abstract, investigated the accuracy of 6 different CVR scores (FRS-lipids, FRS-BMI, RRS, QRISK2, and SCORE) in a cohort of 130 RA patients, age 40–75 screened for the presence of carotid plaque (50% had plaque on ultrasound examination), and found that the presence of plaque was higher in patients classified as moderate/high risk using ASCVD and QRISK2 scores (53).

The inclusion of RA-related indicators, such as disease activity and duration, patient disability, and daily prednisolone use, improved the classification of RA patients based on CVR assessment in addition to the use of traditional CVR factors in a large study from the Consortium of Rheumatology Researchers of North America registry, which requires further validation (54).

Although patients with SLE are usually younger than patients with other ARDs, the analysis of several modified version of FRS revealed that a modified FRS, in which each item was multiplied by 2, was more accurate in predicting coronary artery disease in a large cohort of 904 SLE patients (55). In another study, five conventional stratification tools underestimated the CVR associated with SLE by 50%, and three “lupus adapted” scores (the QRISK3, modified FRS, and modified SCORE risk scores) misclassified 25% of the SLE patients whose CVD risk was defined by the presence of atherosclerotic plaque detected by ultrasound (56).

No validated CVR scores have been used in JIA, although a few studies investigated the prevalence of increased blood pressure in children and young people with JIA compared to healthy controls. Both systolic and diastolic blood pressure were increased in prepubertal children with oligo- and polyarticular JIA (57) as well as in another cohort of 45 children with JIA (58), while HDL-cholesterol levels were lower in a separate study involving 51 JIA patients (59), when compared to age and sex-matched healthy controls.

Despite limitations of QRISK scores in assessing risk in younger populations, the newest version of QRISK score-QRISK3 performed better than the FRS and QRISK2 score in terms of identifying significantly more SLE patients with an increased 10-year risk for CVD, and this risk stratification correlated with markers for endothelial dysfunction and with patients' systolic blood pressure (60). In addition, QRISK3 score also classified a higher number of SLE patients as at risk for developing CVD than the FRS and ASCVD scores (61). A comparison between the FRS and ACC/AHA ASCVD (2013 version) found that 7 and 11.5% of SLE and RA patients, respectively, had discordant CVR scores, which were influenced by disease duration, hsCRP levels, African-American race, diabetes, current use of anti-hypertensive medication, higher age, and higher systolic blood pressure (62). To our knowledge, there are no studies investigating the performance of CVR stratification tools in JSLE or other ARDs with childhood onset.

## Should CVR Stratification Tools for Young Patients With ARD Include Other Biomarkers?

Although there is no consensus regarding the best risk stratification tool for use in young patients with ARDs, some studies investigated correlations between traditional and non-traditional CVR factors and CVD outcomes in younger patients with ARDs.

Patients with JIA had decreased FMD measured at the brachial artery and increased carotid IMT (CIMT compared to matched controls). The systemic JIA phenotype was characterized by the most pronounced abnormalities, with CIMT increase correlating with age, BMI, blood pressure, disease activity, and corticosteroids use (63). A recent study exploring lipid abnormalities in JIA found them present in 83.3% of patients, with low HDL-cholesterol levels being the most common (64). Similarly, systemic JIA was associated more frequently with abnormal LDL-cholesterol and non-HDL-cholesterol, as well as apolipoprotein B levels. Biologic treatment was associated with increased apolipoprotein A1 levels, which correlated negatively with the erythrocyte sedimentation rate (ESR).

A study evaluating 54 adolescents with JSLE found that various risk factors, such as hypertension, elevated triglycerides, apolipoprotein B, hemoglobin A1c and insulin levels, in addition to non-traditional CVR, such as elevated homocysteine and fibrinogen, were altered in adolescents with JSLE compared to matched healthy controls (65). In addition, vascular dynamic testing found increased arterial stiffness measures, central pulse wave velocity and characteristic impedance in JSLE. In multivariate analysis, LDL-cholesterol correlated positively with cumulative prednisone dose and negatively with hydroxychloroquine treatment, providing evidence that both disease and treatment can influence CVD risk.

Recent research has identified lipid biomarker abnormalities in JSLE using an in-depth metabolomic profiling including 230 metabolites, which enabled patient stratification in two groups, one with a pro-atherogenic and one with an athero-protective lipid profile (66). The apolipoprotein-B:A1 ratio distinguished between the two JSLE patient groups with high specificity (96.2%) and sensitivity (96.7%). The lipid signatures identified in the JSLE patients group with an atherogenic lipid profile overlapped significantly with lipid biomarkers associated with sub-clinical atherosclerosis in an independent adult SLE cohort, providing evidence that apolipoprotein-B:A1 ratio could be useful for CVR stratification in JSLE. Interestingly, these lipid signatures were associated with changes in gene expression in T cells, which are now recognized important players in the pathogenesis of atherosclerosis (67).

Lipid abnormalities in female adolescents (10–19 years old) with JSLE have been targeted by a dietary intervention which led to an improved lipid profile in the active treatment group (68), providing evidence that diet could be a suitable strategy for improving JSLE CVR profile.

## Discussion

Despite evidence of progress achieved in identifying CVR biomarkers in young patients with ARDs, there are currently no validated CVR stratification tools recommended for use in these patients, and therefore an unmet patient need to identify and manage CVR earlier in life.

In our opinion, testing of available CVR tools that perform adequately in younger populations (such as QRISK-lifetime CVR score or the PDAY score) or considering clinical history relevant for these patients' background of chronic inflammatory conditions and/or stratifying patients based on inflammatory markers and/or use of steroid treatment (such as QRISK3, RSS) could be a good starting point to identify which stratification tools perform best in children and adolescents with ARDs.

The next step will be to include previously tested or new CVR biomarkers to investigate whether the prediction power of the available scores can be improved. More investment in early detection of atherosclerotic lesions in young patients with ARDs using vascular scans/cardiac MRI or other less-invasive validated measures would facilitate adequate testing of the performance of various CVR stratification tools in real-life.

The greatest investment should be dedicated to following young patients with childhood onset ARDs into adulthood to collect real-life data related to prevalence of CVR events through linking pediatric and adult registries. In order to address the unmet patient need, well-designed clinical trials of various CVR interventions should be performed to investigate if young patients with ARDs have been stratified adequately for CVR interventions using the risk scores proposed, as well as to assess the impact of various interventions on CVD outcomes.

## Author Contributions

CC performed the literature review and wrote the first draft of the manuscript. All authors reviewed the manuscript and approved the final version.

## Funding

This work was supported by grants from the NIHR UCLH Biomedical Research Centre grant BRC772/III/EJ/101350, BRC773/III/CC/101350, and Lupus UK and was performed within the Centre for Adolescent Rheumatology Versus Arthritis at UCL. UCLH and GOSH supported by grants from Versus Arthritis (21593 and 20164), GOSCC, and the NIHR-Biomedical Research Centres at both GOSH and UCLH. The views expressed are those of the authors and not necessarily those of the NHS, the NIHR or the Department of Health.

## Conflict of Interest

The authors declare that the research was conducted in the absence of any commercial or financial relationships that could be construed as a potential conflict of interest.

## Publisher's Note

All claims expressed in this article are solely those of the authors and do not necessarily represent those of their affiliated organizations, or those of the publisher, the editors and the reviewers. Any product that may be evaluated in this article, or claim that may be made by its manufacturer, is not guaranteed or endorsed by the publisher.
